# Germline c.1A>C heterozygous pathogenic variant in *SDHA* reported for the first time in a young adult with a gastric gastrointestinal stromal tumour (GIST): a case report

**DOI:** 10.1186/s13053-019-0124-6

**Published:** 2019-08-09

**Authors:** Sergio Carrera, Elena Beristain, Aintzane Sancho, Eluska Iruarrizaga, Pilar Rivero, Juan Manuel Mañe, Guillermo López Vivanco

**Affiliations:** 1 Hereditary Cancer Genetic Counseling Unit- Medical Oncology Department, Cruces University Hospital, Plaza de Cruces s/n., 48903 Baracaldo, Bizkaia Spain; 2 Molecular Genetics Laboratory, Araba University Hospital, Vitoria, Spain; 3 Medical Oncology Department, Cruces University Hospital, Baracaldo, Spain

**Keywords:** GIST, Hereditary, *SDH*, Paraganglioma, *KIT*, *PDGFRα*, Cancer genetic counseling

## Abstract

**Background:**

Gastrointestinal stromal tumors (GISTs) represent the most frequent mesenchymal tumor of the gastrointestinal tract. Less than 5% of them seem to be hereditary, being succinate dehydrogenase complex (*SDHx*) deficient disorders and neurofibromatosis type 1 the more related inherited conditions. Wild type (WT) *KIT* and *PDGFRα* GISTs constitute a clue for a hypothetical underlying germline condition.

**Case presentation:**

We present a case of a 20 years old female diagnosed of a gastric WT GIST who developed hepatic metastases during her clinical course. No significant or typical phenotypic features suggestive of a specific syndrome were detected by physical examination. Also, her family history seemed to be irrelevant, since no other cases of GISTs, paragangliomas or pheochromocytomas were reported. Her paternal grandfather died as a consequence of a pituitary adenoma. In light of the age of tumor presentation and somatic features of gastric GIST, we performed genetic testing of *SDHx* genes. Analysis obtained from peripheral blood sample revealed the presence, in heterozygous state, of the c.1A > C; p.(Met1?) pathogenic variant in the *SDHA*.

**Conclusions:**

To the best of our knowledge, this is the first published report in which the c.1A > C; p.(Met1?) pathogenic variant in the *SDHA* is associated with a GIST. *SDHA* pathogenic variants increase the risk of paraganglioma, pheochromocytoma, GIST, pituitary adenoma and renal cancer in an autosomal dominant inherited condition named paraganglioma syndrome type 5. The absence of family history of tumors in *SDHA* pathogenic variants carriers could be related to its low penetrance. All patients diagnosed with WT GISTs should be referred to a hereditary cancer genetic counseling unit regardless of the age at presentation or the absence of a suspicious family history.

## Background

Gastrointestinal stromal tumors (GISTs) constitute the most frequent mesenchymal tumor of the gastrointestinal tract [1]. About 70% of GISTs develop in the stomach and 20% in small intestine, being the rest of the gastrointestinal tract or other abdominal organs less frequently affected [2]. The mean age at presentation of GISTs is 60 years old, but in rare cases affects young adults and children [3]. These tumors are driven mostly by CD117 (*KIT*) and platelet-derived growth factor receptor alpha (*PDGFRα*) gain of function mutations [4] but, about a 15% [5] are *KIT* and *PDGFRα* wild type (WT). These WT GISTs are predominantly somatic succinate dehydrogenase (*SDH*) deficient and have a predilection for gastric location [6]. The younger the patient diagnosed with a GIST is, the higher is the probability of harboring a SDH-deficient tumor [7].

Less than 5% of GISTs have an underlying hereditary or syndrome related condition, with neurofibromatosis type 1 (NF1) the most frequent related disease [8]. We can classify these hereditary GISTs in two main groups: SDH-competent and SDH-deficient GISTs [9]. The first group includes *KIT*-mutated and *PDGFRα*-mutated syndromic GISTs, with germline mutations of *KIT*, and *PDGFRα* respectively. Until now few cases of families harboring *KIT* or *PDGFRα* germline mutations have been published [10, 11]. NF1 constitutes the main hereditary entity related to GIST in the SDH-competent group [12] and it is estimated that about 7% of NF1 patients will develop this tumor [13]. GISTs constitute the main gastrointestinal feature in NF1 affected patients. In NF1 GISTs are almost always *KIT* and *PDFGRα* WT, they tend to be multifocal and have predilection for the small bowel [14]. The second group corresponds to the SDH-deficient GISTs and they are related to germline pathogenic variants in succinate dehydrogenase complex (*SDHx*) genes or *SDHC* promoter hypermethylation [15]. In WT GISTs related to *SDHx*, the stomach is the main organ of presentation, usually in the gastric antrum, as multifocal disease of epithelioid variant [16]. SDH-deficiency represents the most important WT GIST subgroup [17]. These WT GISTs tumors have a higher rate of lymphovascular invasion and liver metastases and they are not expected to respond to tyrosine kinase inhibitor imatinib as they lack *KIT* or *PDGFRα* oncogenic mutations [18]. Paradoxically these *SDHx* deficient GIST tumors seem to be more indolent even in the presence of advanced disease compared to *KIT* or *PDGFRα* dependent GISTs [19].

The *SDHx* genes or mitochondrial complex 2 (*SDHA, SDHB, SDHC, SDHD*) encode the subunits of the mitochondrial SDH enzyme, that are essential in the conversion of succinate to fumarate in the Krebs cycle (tricarboxylic acid or citric acid cycle), playing a critical role in mitochondrial respiratory and metabolic functions [20]. If any component of this complex is affected, the entire SDH complex becomes unstable and SDHB immunohistochemistry (IHC) becomes negative because it is rapidly degraded in the cytoplasm. As a consequence, loss of expression of SDHB in a tumor specimen could be used as a suspicion of a germline *SDHx* mutation [21]. SDH deficiency generates an accumulation of intracellular succinate which causes activation of different cellular pathways, favoring tumorigenesis in last instance [22].

*SDHx* germline mutations have been associated to different kind of tumors, mainly paraganglioma and pheochromocytoma. *SDHA* (5p15.33) heterozygous pathogenic variants are related to an autosomal dominant inherited condition named paraganglioma syndrome type 5 (PGL5, OMIM 614165) associated to an increase in the risk of developing paragangliomas, pheochromocytomas, GIST and pituitary adenomas [23]. The Carney-Stratakis syndrome (CSS) also known as GIST-paraganglioma dyad, (OMIM 606864) characterized by the presence of paraganglioma and GIST in the same individual, is related to germline mutations in *SDHB*, *SDHC* and *SDHD*, but its association with *SDHA* pathogenic mutations has been rarely recognized [24]. Carney triad (CT, OMIM 604287), first described in 1977, is characterized by the presence of gastric GISTs, paragangliomas and pulmonary chondromas, it’s almost always not inherited and it’s related to *SDHC* promoter hypermethylation [25]. Lastly, homozygous and compound heterozygous mutations in the *SDHA* have been occasionally related to the Leigh syndrome [26], a rare recessive disease characterized by neurodegenerative mitochondrial encephalomyopathy that becomes apparent mostly in the first year of life.

## Case presentation

A 20 year old female was referred to our Medical Oncology department after a gastric antrum GIST resection. A subtotal gastrectomy revealed a pathologic specimen compatible with an epithelioid GIST variant localised to the muscularis propia. The diameter of the tumor was 1.8 cm and the mitotic count rate was of 18 per 50 HPF (high-power field). IHC assays revealed that the cells presented a strong positive expression of CD117 (KIT) and DOG1, which supported a GIST diagnosis. The tumor was classified as a pT1pN0 (0/1) with a high mitotic rate - stage II – according to the eighth edition of TNM classification. Armed Forces Institute of Pathology (AFIP) criteria calculate the risk of this tumor relapsing and/or progressing as zero, due to the low number of published cases. The mutational analysis revealed that the tumor was *KIT* and *PDGFRα* WT. After complete resection, a whole body computed tomography (CT) scan with contrast was performed, revealing no signs of metastases. Two years after the initial diagnosis, during the follow-up, a CT scan, a magnetic resonance imaging (MRI) of the liver and a positron emission tomography (PET) were performed. These procedures revealed the presence of multiple hepatic metastases. After confirmation of resectability, she underwent surgical resection of the hepatic metastases and postsurgical image studies confirmed no evidence of disease so, in accordance with current medical evidence in patients with complete resection of WT GIST, adjuvant treatment with imatinib was not delivered. At present, the patient is 26 years old and she has no evidence of active disease.

Because of the age at presentation and the molecular features of her tumor, the patient was sent to our Hereditary Cancer Genetic Counseling Unit for further investigations. Complete physical examination was irrelevant and no typical phenotypic features suggestive of a specific syndrome were detected. The family history of the patient seemed to be unremarkable: she has a 19 years old healthy brother. Her parents, 51 years old, had no history of any diseases. The maternal family history was anodyne but the paternal family history included a grandfather who had died at the age of 52 as a consequence of a pituitary adenoma. He was diagnosed when he was 30 years of age and he received radiotherapy treatment. There was no history of other tumors (paraganglioma, pheochromocytoma, GIST or pulmonary chondromas) or other significant diseases in maternal or paternal lines.

On the basis of the described molecular features of diagnosed GIST, age at presentation and in a spite of an apparently irrelevant family history, we decided to perform *SDHx* germline analysis in peripheral blood, after performing pretest counseling and obtaining informed consent.

## Methodology

Analysis of exons 9, 11, 13 and 17 of *KIT* and exons 12, 14 and 18 of *PDGFRα* were performed in tumor samples using amplification of the exons of interest by polymerase chain reaction (PCR) followed by direct sequencing (Sanger method) of amplification products.

Sequence analysis of coding exons and flanking intronic regions of *SDHD* (NM_003002.2), *SDHB* (NM_003000.2), *SDHC* (NM_003001.3) and *SDHA* (NM_004168.2) was undertaken in germline DNA using standard PCR and direct sequencing reactions, avoiding *SDHC* and *SDHA* pseudogenes (BigDye v3.1, Applied Biosystems, Foster City, CA). (Primer sequences and PCR conditions are available on request). Sequencing reactions were analyzed using a 3500 Genetic Analyzer and the data were processed by Sequencing Analysis and Variant Reporter software (all from Applied Biosystems), Detection of large rearrangements in these genes was performed by multiplex ligation-dependent probe amplification (MLPA) analysis, using a commercially available kit (SALSA MLPA P226 kit, MRC-Holland, Amsterdam), according to the manufacturer’s instructions.

## Results

**Fig. 1:**
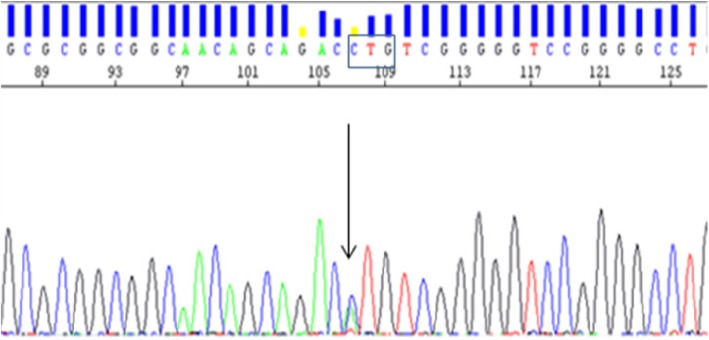
Electropherogram showing the *SDHA* c.1A > C variant

Germline genetic analysis revealed the presence of a heterozygous c.1A > C; p.(Met1?) pathogenic variant in the *SDHA*. This missense variant is predicted to be pathogenic [27, 28] because it affects to the initiation codon AUG (methionine), which is responsible for translation initiation from the messenger RNA (mRNA). This variant is absent in gnomAD (genome aggregation database). Figure 1 shows an electropherogram with the *SDHA* c.1A > C variant.

**Fig. 2:**
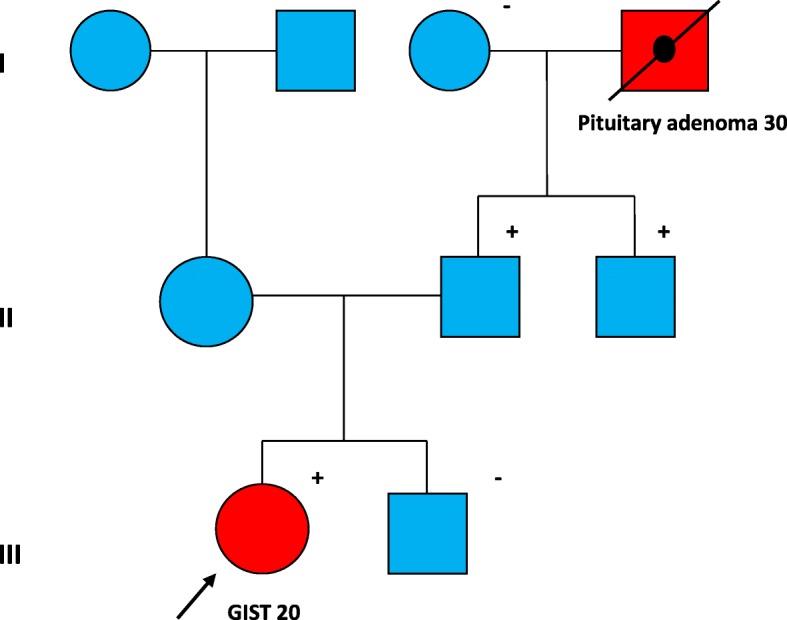
Family pedigree. Unaffected female: blue circle. Unaffected male: blue square. Affected female: red circle. Affected male: red square Plus (+) and minus (−) signs represent carrier status of tested family members

Subsequently, we performed predictive tests to her relatives. Her brother was found not to be a carrier of the variant; her father and paternal uncle were carriers (confirming paternal inheritance) and her paternal grandmother was also negative for this variant. These results suggest that the paternal grandfather, who died as a consequence of a pituitary adenoma, could be obligate carrier of the c.1A > C; p.(Met1?) pathogenic variant in the *SDHA*. Figure 2 shows the family pedigree.

## Discussion and conclusions

An exhaustive review of databases and the medical literature was undertaken in order to assess the pathogenicity of this variant. The c.1A > C; p.(Met1?) variant has previously been reported in a patient diagnosed with Leigh syndrome, in which genetic analysis revealed the presence of a compound heterozygote mutation in the *SDHA*. Second allele mutation, in *trans* with the other *SDHA* pathogenic variant, corresponded to a heterozygous A to C substitution which changed the methionine translation initiation codon to a leucine. Additional functional studies confirmed a quantitative decrease and instability of the corresponding mRNA, which supports its causality [28]. On the basis of this study, ClinVar database (http://www.ncbi.nlm.nih.gov/clinvar/) describes the c.1A > C; p. (Met1?) variant in *SDHA* as pathogenic [29].

In another recent report which includes 972 patients with paraganglioma-pheochromocytoma [30], a 66 year old patient diagnosed of thoracic paraganglioma, with negative family history of other malignancies, carried the c.1A > C pathogenic variant in the *SDHA*. Another germline pathogenic variant in the same codon (c.2 T > C) has been reported in a 23 year old patient with a WT GIST and a renal chromophobe cell tumor [31].

*SDHA* heterozygous pathogenic variants are related to a different spectrum of tumours. Germline *SDHA* variants have been quite recently reported to be associated to paragangliomas and the disease penetrance among carriers is estimated to be low [32]. As a consequence the family history of the carriers can go unnoticed. PGL5 is associated with an increase in the risk of developing paragangliomas, pheochromocytomas, GISTs and pituitary adenomas, and it’s compatible with our clinical suspicion on the basis of this patient’s GIST, her paternal grandfather pituitary adenoma [33] and the co-segregation of the c.1A > C pathogenic variant in the *SDHA* gene with the disease. The CSS, characterized by the presence of paraganglioma and GIST in the same individual is principally related to germline mutations in *SDHB*, *SDHC* and *SDHD*, but its association with *SDHA* pathogenic mutations is more exceptional. Strictly, we cannot rule out a hypothetical clinical diagnosis of CSS in our patient, but at the moment no paragangliomas have been detected by radiological studies. We also emphasize that her paternal grandfather (obligate carrier of *SDHA* pathogenic variant) died as a consequence of a pituitary adenoma, which is also associated with *SDHA* pathogenic variants, but it’s not a typical feature of CSS. *SDHx* deficiency related diseases and their phenotypes frequently overlap. In fact, currently, in an era in which genetic analyses are more accurate and performed more extensively, patients with a previous clinical diagnosis of CT can show *SDHx* mutations [34]. The clinical course of our patient and of the c.1A > C; p.(Met1?) *SDHA* pathogenic variant carriers in her paternal family could help define better their clinical diagnoses in the spectrum of *SDHA* inherited related conditions, but we must take into consideration that *SDHA* variants have a reduced disease penetrance compared to other components of *SDHx* complex, especially *SDHB* and *SDHD* [35].

GISTs constitute the most frequent mesenchymal tumor of the gastrointestinal tract [1]. WT GISTs are predominantly somatic SDH-deficient and have predilection for gastric location [6]. The younger the diagnosis of GIST is, the higher the probability of harboring a SDH-deficient tumor [7]. As previously mentioned, these SDH-deficient GISTs seem to be more indolent even in the presence of advanced disease compared to *KIT* or *PDGFRα* onco-addicted GISTs [19]. Our patient represents a clear example of the more indolent biological behaviour of the SDHx-deficient GISTs in the context of advanced disease: having survived six years since her initial diagnosis with no evidence of recurrent disease. In addition, the clinical case reflects the need for further studies in those GISTs with an early age at presentation, even in the absence of clear family history or other pathognomonic features.

To the best of our knowledge this is the first published report in which the c.1A > C; p.(Met1?) pathogenic variation in the *SDHA* seems to be associated to a gastric GIST in the context of PGL5. We must emphasize that *SDH* screening in patients with GIST, especially in those diagnosed at early ages, should be undertaken. All patients diagnosed of WT GISTs should be referred to a hereditary cancer genetic counseling unit.

## Data Availability

Not applicable.

## Abbreviations

CSS: Carney-Stratakis syndrome
CT: Carney triad
GIST: Gastrointestinal stromal tumor
KIT: KIT proto-oncogene receptor tyrosine kinase
PDGFRα: Platelet-derived growth factor receptor alpha
PGL: Paraganglioma
SDH: Succinate dehydrogenase
WT: Wild type
